# Supplementary material for: Continuation of education after marriage and its relationship with professional maternal healthcare utilization among young adult women in Bangladesh
Source: PLoS One. 2024 Dec 31;19(12):e0316117. doi: 10.1371/journal.pone.0316117 (PMC11687904; doi:10.1371/journal.pone.0316117)
Supplement: Attachment — Submitted filename: Response to reviewer.docx [file pone.0316117.s001.docx]

29 September, 2024

Md. Moyazzem Hossain
Academic Editor
Plos One

**Re: Manuscript Number** PONE-D-24-13879

Dear Prof. Md. Moyazzem Hossain,

Thank you for considering and giving us the chance to submit the revision of our manuscript, **Continuation of education after marriage and its relationship with professional maternal healthcare utilization among young adult women in Bangladesh,** for possible publication in Plos One. We revised the manuscript based on the editor and reviewer’s feedback. Their useful and productive comments helped us improve the clarity and quality of the manuscript. Using the track change option, we have changed the text in the manuscript. It is to mention that this manuscript has not been published or presented elsewhere in part or in entirety and is not under consideration by another journal. We have read and understood the journal's policies, and we believe that neither the manuscript nor the study violates any of these. The authors have no conflicts of interest to declare. As a corresponding author, I confirm full access to all aspects of the research and writing process, and final responsibility for the paper.

We hope that the revisions are satisfactory in addressing issues raised by the reviewers and look forward to hearing your decision about this article.

Yours sincerely,

Corresponding Author

**Editor’s comment**

1. Revise the manuscript carefully based on the reviewer's comments and follow the journal style.

**Response:** Thank you for giving us the opportunity to revise the manuscript. We have carefully accommodate all the issues while revising the manuscript.

2. In Figure 1, it is suggested to show the complete percentages of ANC visits i.e., presents all categories.

**Response:** Thank you. We have revised it accordingly.

3. In Figure 2, it is recommended to add the scale and direction.

**Response:** Thank you. We have added the scale and direction in the revised version of figure 2.

4. Add strengths and limitations as a separate section.

**Response:** Thank you. We have separated this section in text.

5. Specify which goals of SDGs will be attained.

**Response:** Thank you. We have specified in the conclusion section of the text that SDG 3 will be attained.

Journal requirements:

When submitting your revision, we need you to address these additional requirements.

1. Please ensure that your manuscript meets PLOS ONE's style requirements, including those for file naming. The PLOS ONE style templates can be found at

https://journals.plos.org/plosone/s/file?id=wjVg/PLOSOne_formatting_sample_main_body.pdf and

<https://journals.plos.org/plosone/s/file?id=ba62/PLOSOne_formatting_sample_title_authors_affiliations.pdf>

**Response:** We have revised the manuscript following PLOS ONE’s guidelines for preparing manuscript.

2. Thank you for stating the following in your Competing Interests section:

“None declared.”

Please complete your Competing Interests on the online submission form to state any Competing Interests. If you have no competing interests, please state "The authors have declared that no competing interests exist.", as detailed online in our guide for authors at http://journals.plos.org/plosone/s/submit-now

 This information should be included in your cover letter; we will change the online submission form on your behalf.

**Response:** Thank you. We did it.

3. Your ethics statement should only appear in the Methods section of your manuscript. If your ethics statement is written in any section besides the Methods, please move it to the Methods section and delete it from any other section. Please ensure that your ethics statement is included in your manuscript, as the ethics statement entered into the online submission form will not be published alongside your manuscript.

**Response:** Thank you. We have moved the ethics in the method section according to the journal’s requirement.

4. We note that Figure 2 in your submission contain [map/satellite] images which may be copyrighted. All PLOS content is published under the Creative Commons Attribution License (CC BY 4.0), which means that the manuscript, images, and Supporting Information files will be freely available online, and any third party is permitted to access, download, copy, distribute, and use these materials in any way, even commercially, with proper attribution. For these reasons, we cannot publish previously copyrighted maps or satellite images created using proprietary data, such as Google software (Google Maps, Street View, and Earth). For more information, see our copyright guidelines: http://journals.plos.org/plosone/s/licenses-and-copyright.

We require you to either (1) present written permission from the copyright holder to publish these figures specifically under the CC BY 4.0 license, or (2) remove the figures from your submission:

a. You may seek permission from the original copyright holder of Figure 2 to publish the content specifically under the CC BY 4.0 license.

We recommend that you contact the original copyright holder with the Content Permission Form (http://journals.plos.org/plosone/s/file?id=7c09/content-permission-form.pdf) and the following text:

“I request permission for the open-access journal PLOS ONE to publish XXX under the Creative Commons Attribution License (CCAL) CC BY 4.0 (http://creativecommons.org/licenses/by/4.0/). Please be aware that this license allows unrestricted use and distribution, even commercially, by third parties. Please reply and provide explicit written permission to publish XXX under a CC BY license and complete the attached form.”

Please upload the completed Content Permission Form or other proof of granted permissions as an "Other" file with your submission.

In the figure caption of the copyrighted figure, please include the following text: “Reprinted from [ref] under a CC BY license, with permission from [name of publisher], original copyright [original copyright year].”

b. If you are unable to obtain permission from the original copyright holder to publish these figures under the CC BY 4.0 license or if the copyright holder’s requirements are incompatible with the CC BY 4.0 license, please either i) remove the figure or ii) supply a replacement figure that complies with the CC BY 4.0 license. Please check copyright information on all replacement figures and update the figure caption with source information. If applicable, please specify in the figure caption text when a figure is similar but not identical to the original image and is therefore for illustrative purposes only.

The following resources for replacing copyrighted map figures may be helpful:

USGS National Map Viewer (public domain): http://viewer.nationalmap.gov/viewer/

The Gateway to Astronaut Photography of Earth (public domain): http://eol.jsc.nasa.gov/sseop/clickmap/

Maps at the CIA (public domain): https://www.cia.gov/library/publications/the-world-factbook/index.html and https://www.cia.gov/library/publications/cia-maps-publications/index.html

NASA Earth Observatory (public domain): http://earthobservatory.nasa.gov/

Landsat: http://landsat.visibleearth.nasa.gov/

USGS EROS (Earth Resources Observatory and Science (EROS) Center) (public domain): http://eros.usgs.gov/#

Natural Earth (public domain): http://www.naturalearthdata.com/

**Response:** We prepared Figure 2 by using ggplot2 package and sf package in R.

**Reviewer**

**Reviewer #1: Many thanks for inviting me to review this paper, and I am extremely sorry for the delay in submitting my revision. I read this paper with a lot of interest and care; however, I found several areas that suggest rejection of this paper for publication in PLoS ONE or any other journal.**

**Response:** Thank you very much for your valuable time for reviewing our manuscript.  **My particular concern lies in the association that the author explored. The survey that the authors analyzed collected data on the continuation and discontinuation of education only for women who married early. I request the authors to check the survey reports and relevant questions for details. Therefore, all the samples analyzed were women who married early. Statistics related to age at first marriage in Table 1 also support this, as the reported early marriage rate is 73% compared to the national average early marriage rate of 51%, according to the same survey. However, it is unfortunate that the authors manipulated part of the data by mentioning that 26.8% did not marry early, which is impossible considering the data analysis.**

**Response:** Thank you. We did review the report before beginning the analysis. However, in response to your recommendation, we rechecked the BDHS 2017-18 final report. The survey only asked women who were studying just before the marriage the question about continuation or discontinuation of education. Exact wording in the survey questionnaire was: “Were you studying or attending school just before you got married?" Response option: “Yes or No." If yes, then they were asked about how long they continued. Please see **Appendix G, Page No. 361** of the final report of BHDS 2017-18.

We found no reference in the report that data on educational continuation/discontinuation were only obtained for women who got married early (before the age of 18). Rather, we observed in **Table 4.10 on page 52** of the study that 80.5% of women who marry before the age of 18 quit their education, compared to 62.8% and 45% for women who marry between the ages of 18 and 20 and 21 and older, respectively. If the BDHS only collected information for women who married before the age of 18, how did they categorize such proportions?

It's disappointing that the reviewer accused us of manipulating data. We would be pleased to offer the DO file for our analysis at any time.

**In addition to this issue, considering the early marriage sample, which is mostly from the lower quintile, as representative of the total sample is not logical. In such cases, the main issue is whether these women had the knowledge and decision-making autonomy to access healthcare services rather than the continuation of education. The authors’ analysis here seems to focus on a minor issue while hiding a more important one.**

**Response:** Thank you. First of all, data were not collected for women who got married early but rather collected based on their study or attendance at school just before marriage. The eligible women for this current study were young adults (aged 15–29 years), currently married, and who had at least one live birth in the previous three years of the survey.  Our data also shows that 11.8% and 16.7% were from the poorest and poor wealth quintiles.

Ample of studies  across the globe, including researchers from high-income countries, have also documented that household decision-making autonomy was found to be associated with maternal and child healthcare utilization. A very few examples are given below:

Story WT, Burgard SA. Couples' reports of household decision-making and the utilization of maternal health services in Bangladesh. Soc Sci Med. 2012 Dec;75(12):2403-11. doi: 10.1016/j.socscimed.2012.09.017. Epub 2012 Sep 26. PMID: 23068556; PMCID: PMC3523098.

Pokhrel S, Sauerborn R. Household decision-making on child health care in developing countries: the case of Nepal. Health Policy Plan. 2004 Jul;19(4):218-33. doi: 10.1093/heapol/czh027. PMID: 15208278.

Gebeyehu NA, Gelaw KA, Lake EA, Adela GA, Tegegne KD, Shewangashaw NE. Women decision-making autonomy on maternal health service and associated factors in low- and middle-income countries: Systematic review and meta-analysis. *Women’s Health*. 2022;18. doi:[10.1177/17455057221122618](https://doi.org/10.1177/17455057221122618)

Xiaohui Hou, Ning Ma, The effect of women’s decision-making power on maternal health services uptake: evidence from Pakistan, *Health Policy and Planning*, Volume 28, Issue 2, March 2013, Pages 176–184, <https://doi.org/10.1093/heapol/czs042>

The household decision-making power/autonomy was only included as covariates based on earlier studies. Yes, there are a lot of other important issues that affect maternal healthcare utilization; however, we should not ignore any minor issues that could have potential relationships with healthcare utilization. Since education of mothers is a powerful factor in seeking healthcare, therefore, we expected that women who did not continue their education or not complete their education are less likely to utilize healthcare facilities. Therefore, we conducted this study.

**Moreover, according to the government of Bangladesh, the recommended number of ANC visits is four, although WHO changed it to six to eight visits as the global recommendation. As such, it is completely misleading if the authors consider eight ANC visits as effective for Bangladesh. Additionally, the authors generated several variables, and a few of them are completely misleading, like media access categorized as "no access" and "some access." Is it possible to find people who are not connected in any way with radio, television, or newspapers that were used to generate this variable? Moreover, I am not sure how it is logical to say "some access" if respondents reported they accessed newspapers, radio, and television. Moreover, adding this group, i.e., all three access types, with the group that reported access to one or two types and classifying them together is also very problematic. It seems the authors compared a banana with an apple, which is funny instead.**

**Response:** Thank you. Although WHO recommended eight visits for complete ANC visits in 2016, Bangladesh country recommendations continue to promote four or more ANC visits. The goal of this cross-sectional study was not to evaluate the effectiveness of eight ANC visits in Bangladesh; rather, we wanted to see how mothers' continued education was connected with greater or equivalent to eight ANC, which the government could implement in the future.

In terms of media access, I agree that it's difficult to imagine people without access to radio, TV, or newspapers. As shown on page 31 of the BDHS 2017-18 final report, over two-fifths (44%) of women are not frequently exposed to any of these kinds of media; just 3.2% listen to radio, 55% watch TV, and 2.1% read a newspaper once a week (Table 3.4, page 37).  Although we may cite a number of studies in which the authors constructed media exposure, as we did here because you have objections about such categories, we present these media individually, along with their original classification, and amend our analysis.

**The authors also generated some funny and useless maps. For instance, the first two maps regarding ANC in Figure 2 are mutually exclusive; however, they never mentioned this. Moreover, the authors used the range of their reported prevalence as the label of the maps. However, what is the explanation for this? It seems the maps were created without any statistical knowledge.**

**Response:** Thank you. In figure 2, ≥4 ANC and ≥8 ANC are not mutually exclusive. Respondents who took ≥8 ANC were also included in the category of ≥4 ANC. We removed the label of the reported prevalence, and as suggested by the academic editor, we have included scale and direction to the figure.

**Moreover, consider a typical scenario where a woman gets married at age 14 and stops her education, and the index birth analyzed occurred at age 24. Statistically, is it logical to associate the age at marriage with healthcare service access in such a case?**

**Response:** Thank you. In this regard, cross-sectional study designs are unhelpful. Of course, this study design has limits, but it serves as a foundation for future research and lays the groundwork for investigations into cause-and-effect linkages.

**Although I agree that if you input them into statistical software without proper statistical knowledge, you will definitely get results. This is what happened in your case. Summarizing these, the analysis done is completely useless and WRONG, although the authors did a moderate job in writing the paper with follow-up.**

**Response:** Thank you. We will be pleased to share our Stata DO file anytime for cross-checking the results .  However, we appreciate that you at least you find something moderately good in the manuscript.

**I believe the authors are more interested in publishing their paper rather than considering the scientific value and justification of their paper. This practice is increasingly prevalent among authors in LMICs, including Bangladesh. I request the authors to change their motivation to focus on scientific value rather than merely publication. PLEASE DO NOT WRITE SUCH MEANINGLESS PAPERS. This is never done by a genuine academician.**

**Response:** Thank you. Publishing articles with no scientific merit or justification in LMICs, including Bangladesh, is an excellent observation. We agree with you on this point, but it is not limited to LMICs; we can find similar data in developed countries as well. However, many of these useless studies are published in journals whose editors originate from renowned academic institutions in so-called high-income countries. Their journals also send e-mails inviting authors to submit papers for publication in their journals, which could be a source of income for the journal. Furthermore, so-called high-income countries have constructed a useless ranking system for universities or academic institutions, primarily for business objectives, in which the number of published articles is encouraged. Academic institutions, mainly from LMICs, fall into this trap, encouraging their researchers to publish as many articles as possible. We would appreciate it if you could speak up on this topic. Finally, we would greatly appreciate it if you could suggest or send us some of your publications so that we may learn and develop expertise about doing important scientific papers. Thank you very much.

**Reviewer #2: Dear Authors

Introduction: Expand the literature review in the introduction to provide a more comprehensive background.**

**Response:** Thank you very much. We included some text in the manuscript.

**Response:
Methods: the study design it is not mentioned.**

**Response:** Thank you. We have corrected it in the revised version.

**Expand Future Research Directions: Suggest specific areas for future research**

**Response:** Thank you. We have added a sentence suggesting future area of research in the conclusion section.
